# Dynamic reconstruction of electroencephalogram data using RBF neural networks

**DOI:** 10.3389/fnins.2025.1557763

**Published:** 2025-03-28

**Authors:** Xuan Wang, Congcong Du, Xianjin Ke, Jian Zhang, Zheng Zheng, Yayan Yue, Ming Yu

**Affiliations:** ^1^Department of Neurology, Affiliated Hospital of Jiangsu University, Zhenjiang, Jiangsu, China; ^2^School of Systems Science, Beijing Normal University, Beijing, China

**Keywords:** electroencephalogram, RBF neural networks, age-related analysis, brain dynamics, Particle Swarm Optimization

## Abstract

**Introduction:**

Electroencephalography (EEG) is widely used for analyzing brain activity; however, the nonlinear and nature of EEG signals presents significant challenges for traditional analysis methods. Machine has shown great promise in addressing these limitations. This study proposes a novel approach using Radial Function (RBF) neural networks optimized by Particle Swarm Optimization (PSO) to reconstruct EEG dynamics and extract age-related neural characteristics.

**Methods:**

EEG recordings were collected from 142 participants spanning multiple age groups. Signals were preprocessed through bandpass filtering (1–35 Hz) and Independent Component Analysis (ICA) for artifact removal. neural network was trained on EEG time-series data with PSO employed to optimize model parameters identify fixed points in the reconstructed neural system. Statistical analyses including ANOVA and Kruskal-Wallis tests were performed to assess age-related differences in fixed-point coordinates.

**Results:**

The RBF network demonstrated high accuracy in EEG signal reconstruction across different frequency a normalized root mean square error (NRMSE) of 0.0671 ± 0.0074 and a Pearson correlation coefficient ± 0.0678. Spectral and time-frequency analyses confirmed the model s capability to accurately capture oscillations. Importantly analysis of RBF network fixed-point coordinates revealed distinct age-related.

**Discussion:**

These findings suggest that fixed-point coordinates of RBF networks can serve as quantitative markers aging providing new insights into age-dependent changes in brain dynamics. The proposed method offers computationally efficient and interpretable approach for EEG analysis with potential applications in neurological diagnosis and cognitive research.

## 1 Introduction

Electroencephalography (EEG) stands as a non-invasive, economical, and practical neuroscience research tool (Craik et al., 2019) that captures high-temporal-resolution brain activity by recording postsynaptic potentials of cortical pyramidal neurons (Smailovic and Jelic, 2019). This technology has demonstrated significant value in diagnosing and researching various neuropsychiatric disorders, including epilepsy (Chen and Koubeissi, 2019), Alzheimer’s disease (Ouchani et al., 2021), and depression (Patil et al., 2023; Põld et al., 2023; Tao et al., 2023). Traditional EEG analysis, while valuable, has been limited by its reliance on clinical expertise, consuming significant time and potentially introducing subjective bias (Dong et al., 2021; Gil Ávila et al., 2023; Wiegand et al., 2022). Moreover, the inherent complexity, non-linearity, and non-stationarity of EEG signals (Song and Yoon, 2015) present substantial challenges for accurate interpretation.

Recent years have witnessed remarkable advances in machine learning applications for EEG signal processing and analysis (Hussain et al., 2023; Mirchi et al., 2022; Schmierer et al., 2024). Researchers have successfully implemented various machine learning algorithms, including support vector machines, random forests, and deep learning, to achieve automated feature extraction and classification of EEG data (Chen et al., 2019). These approaches have shown exceptional performance in multiple applications, significantly enhancing diagnostic objectivity and accuracy. Notable achievements include Bhattacharyya et al. (2017) efficient epileptic seizure detection system combining tunable-Q wavelet transform with Least Squares Support Vector Machine (LS-SVM), Li et al. (2024) integrated the Recursive Feature Elimination (RFE) algorithm with the Support Vector Machine (SVM) algorithm and employed a cross-validation method. Their study identified unique energy features in the 4–9 Hz frequency band of coma patients compared to brain-dead patients. The SVM classifier achieved an accuracy of 99.59% (Li et al., 2024) and Hosseinifard et al.’s (2013) high-accuracy depression detection using machine learning classifiers. A recent breakthrough by Amini et al. (2021) demonstrated impressive accuracy rates (89.1%, 85%, and 75%) in distinguishing between Alzheimer’s Disease patients, those with Mild Cognitive Impairment, and healthy controls using convolutional neural networks. These advancements demonstrate machine learning’s capacity to uncover hidden patterns in EEG data, opening new avenues for precise diagnosis and personalized treatment of neuropsychiatric disorders. Recent advances in EEG-based neural modeling have significantly improved our understanding of brain system dynamics, with traditional frequency-domain and time-series analyses now complemented by modern machine learning approaches that enhance predictive capabilities. Studies have explored diverse methodologies, including Markov Chain analysis for EEG pattern identification (Wiemers et al., 2024), deep learning-based EEG feature extraction using CNN, LSTM, and GRU networks (Rivas et al., 2025), and graph neural networks for motor learning and rehabilitation prediction (Han et al., 2025). Additionally, multimodal research integrating EEG, MEG, and fMRI data fusion has expanded insights into neural connectivity and cognitive functions (Baghdadi et al., 2025). Despite these advancements, challenges remain in optimizing EEG models while balancing computational efficiency and interpretability. Deep learning architectures, though powerful, often require large-scale training datasets and extensive computational resources (Wei et al., 2025), limiting their feasibility in real-time applications. In contrast, function approximation methods like Radial Basis Function (RBF) networks offer a practical trade-off between accuracy and computational feasibility, making them particularly suitable for real-time EEG processing.

While machine learning has made remarkable progress in EEG analysis, existing methods face several significant challenges. Traditional approaches rely heavily on manually designed features, potentially missing crucial EEG signal information (Rakhmatulin et al., 2024), while most studies are confined to either temporal or frequency domain analysis (Cai et al., 2020), limiting their ability to fully characterize the complex dynamics of EEG signals. Furthermore, current models often lack interpretability (Yagin et al., 2024), hampering their utility in clinical diagnosis. These limitations underscore the need for novel analytical methods capable of automatic multidimensional feature extraction and accurate characterization of EEG nonlinear dynamics, while maintaining clinical interpretability.

A promising approach emerges from viewing neural electrical activity as a complex dynamical system and reconstructing its governing equations through EEG data analysis. Recent research has demonstrated the unique advantages of RBF neural networks in analyzing complex physiological signals, exemplified by Du et al. (2023) successful differentiation of cardiac characteristics between healthy individuals and heart disease patients. Inspired by these findings, we propose a novel analytical framework based on RBF neural networks for characterizing resting-state brain activity.

## 2 Materials and methods

### 2.1 Ethical statement

This study was conducted in accordance with the principles outlined in the Declaration of Helsinki and was approved by the Ethics Committee of the Affiliated Hospital of Jiangsu University (Approval No. KY2024K1102). All participants were fully informed about the study objectives, procedures, and potential risks before participation. Written informed consent was obtained from all adult participants. For minors (participants under 18 years old), written informed consent was provided by their legal guardians or parents. Participation in the study was entirely voluntary, and all subjects had the right to withdraw at any time without any consequences. The study ensured strict confidentiality and anonymization of all participant data throughout the research process. EEG recordings were conducted in a controlled environment by trained medical professionals to ensure both participant safety and data quality.

### 2.2 Participants

This investigation was conducted at the Affiliated Hospital of Jiangsu University, enrolling patients who underwent health examinations or sought medical attention for symptoms including headache, dizziness, and fever between November 2023 and November 2024, with no evidence of intracranial organic lesions. The study population consisted exclusively of Han Chinese individuals, primarily from Jiangsu Province. Exclusion criteria encompassed: (1) history of neurological disorders including epilepsy, cognitive impairment, Parkinson’s disease, central nervous system infections, craniocerebral trauma, brain tumors, or alcohol intoxication; (2) presence of intracranial organic lesions on current examination; (3) inability to complete EEG examination due to altered consciousness or unstable physical condition; (4) refusal to provide informed consent. A total of 142 subjects met the inclusion criteria and completed the study.

We collected comprehensive data from all participants, including demographic information (gender, date of birth, education level) and clinical data (medical history, head CT/MRI findings). All subjects underwent standardized resting-state EEG examinations, with results confirmed to be within normal parameters.

### 2.3 EEG data acquisition and processing methodology

Electroencephalogram recordings were conducted using a Neurofax EEG-1200C system (Neuroworkbench, Nihon Kohden) with 32-channel capability (including two ECG channels) at 200 Hz sampling frequency. Electrode placement followed the international 10/20 system protocol, with impedances maintained below 5 kΩ. Recordings were performed in a quiet environment, where participants completed a 10 min session of eyes-closed and eyes-open conditions while seated comfortably. Participants were instructed to maintain alertness while minimizing physical movements. All recordings were conducted by a single experienced EEG physician who was blinded to participants’ clinical information. Analysis was restricted to eyes-closed condition data.

**FIGURE 1:**
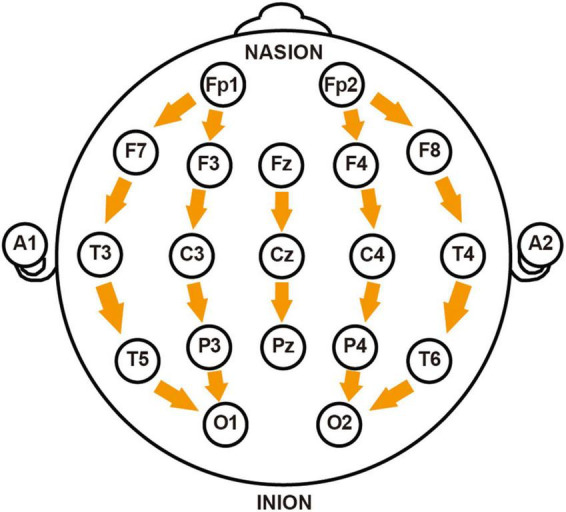
Schematic diagram of longitudinal bipolar electroencephalogram (EEG) electrode montage. The figure illustrates the electrode placement according to the International 10–20 System used in this study, with arrows indicating the direction of longitudinal re-referencing between adjacent electrodes. The montage includes: prefrontal region (Fp1-F7, Fp1-F3, Fp2-F4, Fp2-F8), frontal region (F7-T3, F3-C3, Fz-Cz, F4-C4, F8-T4), central region (T3-T5, C3-P3, Cz-Pz, C4-P4, T4-T6), and occipital region (T5-O1, P3-O1, P4-O2, T6-O2). NASION and INION mark the positions of the nasion and inion, respectively.

Signal processing was performed using Matlab R2021a (The Mathworks, United States). Raw signals underwent bandpass filtering (1–35 Hz) to isolate conventional frequency bands [δ(1–4 Hz), θ(4–8 Hz), α(8–12 Hz), and β(12–30 Hz)] while eliminating noise artifacts. Artifact rejection was implemented using FASTICA algorithm for Independent Component Analysis (ICA). From the 32 recorded channels, 19 key channels were selected (Fp1, Fp2, F7, F3, Fz, F4, F8, T3, C3, Cz, C4, T4, T5, P3, Pz, P4, T6, O1, O2) and re-referenced using a longitudinal bipolar montage, where each channel was referenced to the electrode immediately posterior to it (e.g., Fp1-F3, F3-C3, C3-P3, P3-O1) (illustrated in Figure 1). This anterior-to-posterior bipolar derivation effectively minimized volume conduction effects while enhancing the detection of localized potential differences and improving spatial resolution. Additionally, this montage configuration reduced contamination from common reference artifacts and environmental electrical interference (Babiloni et al., 2020). Ocular and cardiac signals were excluded from the analysis.

### 2.4 EEG data analysis

#### 2.4.1 Step 1: Data preprocessing

This study implements RBF networks to implicitly represent the system’s dynamical equations. The RBF network training process for EEG data consists of three key steps. The first step involves data preprocessing, where Principal Component Analysis (PCA) is applied to reduce 18-dimensional EEG signals to three-dimensional sequences, with 40,000 data points retained per subject for the training set. For an *n*-dimensional continuous dynamical system defined as shown in Equation 1:


(1) $$\dot{X}=F\,\left(X\right),X\in R^{n}$$

The temporal derivatives ${\dot{X}}_{i}\left(i=1,2\right)$ are computed using a fourth-order five-point difference scheme, defined in Equation 2:


$${\text{f}}^{\prime }\left(t_{0}\right)=\frac{1}{12\,h}[f\left(t_{0}-2h\right)-8f\left(t_{0}-h\right)+8f\left(t_{0}+h\right)$$

(2) $$-f\left(t_{0}+2h\right)]+\frac{h^{4}}{30}{\text{f}}^{\left(5\right)}(\xi )$$

The numerical derivative ${\text{f}}^{\prime }$ (t_0_) represents the rate of change in the time series at time point t_0_. For complex systems, we calculate these derivatives from observed time series X_*i*_ using a fourth-order five-point difference method.

#### 2.4.2 Step 2: RBF network training

The second phase involves RBF network training through supervised learning, with an 8:1 split between training and validation sets. The network maps EEG time series X_*i*_ (input) to their corresponding derivatives $\dot{X}$ (output). Training is implemented using MATLAB’s newrbe function, which optimizes network parameters including center vectors (μ), variance of activation function (Σ), the connection weight from hidden neurons to output neurons (W), and hidden layer architecture. The training process employs backpropagation with mean square error minimization through gradient descent optimization. The network features a single hidden layer, with its size adaptively determined by newrbe based on data distribution and accuracy requirements. Connection weights are initialized using uniform random distribution to enhance generalization and avoid local optima. The trained RBF network approximates the function F(X), expressing the system dynamics as formulated in Equation 3:


(3) $$\dot{X}=\mathrm{Network}\,\left(X\right),X\in R^{n}$$

#### 2.4.3 Step 3: Dynamical system analysis

In the third step, numerical methods were implemented to analyze the implicit approximate system defined by Equation 3. The Particle Swarm Optimization (PSO) algorithm (Gad, 2022) was then employed to determine the fixed points ${\bar{X}}_{N}$ of the implicit dynamical system, satisfying (Network (${\bar{X}}_{N}$) = 0). To characterize these fixed points, we introduced small perturbations δ_e_i__ to numerically compute the Jacobian matrix through the perturbative method defined in Equation 4:


(4) $$J_{i}\,\left({\bar{X}}_{N}\right)=\frac{N\,\text{e}\,\mathrm{twork}\,\left({\bar{X}}_{N}+\delta_{e_{i}}\right)-N\,\text{e}\,\mathrm{twork}\,\left({\bar{X}}_{N}\right)}{\delta }$$

The type and stability of the fixed points were determined through eigenvalue analysis of the Jacobian matrix. EEG data exhibiting stable fixed points were selected for subsequent age-related group analysis.

### 2.5 Statistical analysis

All statistical analyses were performed using MATLAB R2021a. Quantitative data are presented as mean ± standard error of mean (SEM), and qualitative variables are described using frequencies and percentages. Error bars in all figures represent SEM. The Shapiro-Wilk test was used to assess data normality (*P* > 0.05 indicating normal distribution). For normally distributed data, comparisons between multiple groups were conducted using one-way ANOVA, while the Kruskal-Wallis test was applied for non-normally distributed data. When significant differences were detected by either ANOVA or Kruskal-Wallis test, *post hoc* multiple comparisons were performed using the Tukey-Kramer method. Multiple group categorical variables were analyzed using Pearson’s chi-square test. All statistical tests were two-tailed, with statistical significance set at *P* < 0.05.

## 3 Results

### 3.1 Subject demographics

This study included 142 participants; demographic characteristics are shown in Table 1. Statistical analysis showed no significant difference in gender distribution between age groups (*p* = 1). The ratio of males to females was approximately 1:1.

**TABLE 1:** Demographic characteristics of study subjects.

Age group (years)	Male *n* (%)	Female *n* (%)	Age (Mean ± SD)	Total *n*
0–10	11 (55.0%)	9 (45.0%)	7.526 ± 1.307	20
10–20	8 (47.1%)	9 (52.9%)	13.444 ± 2.706	17
20–30	5 (50.0%)	5 (50.0%)	24.600 ± 4.061	10
30–40	7 (46.7%)	8 (53.3%)	35.667 ± 2.870	15
40–50	6 (54.5%)	5 (45.5%)	44.455 ± 2.945	11
50–60	13 (52.0%)	12 (48.0)	55.440 ± 2.931	25
60–70	9 (52.9%)	8 (47.1%)	63.824 ± 2.298	17
70–80	9 (52.9%)	8 (47.1%)	73.471 ± 2.211	17
> 80	5 (50.0%)	5 (50.0%)	81.600 ± 2.366	10
Total	74 (52.1%)	68 (47.9%)	43.599 ± 24.789	142

### 3.2 Assessment of RBF network prediction accuracy

To evaluate the RBF neural network’s performance in EEG data modeling, we selected 40,000 data points from each participant’s EEG recordings for network training. Participants were stratified into nine age groups to ensure comprehensive capture of age-specific EEG characteristics. Following training, the model’s predictive capability was evaluated using the remaining data (approximately 5,000 points) as a test set for EEG waveform prediction on previously unseen time series.

**FIGURE 2:**
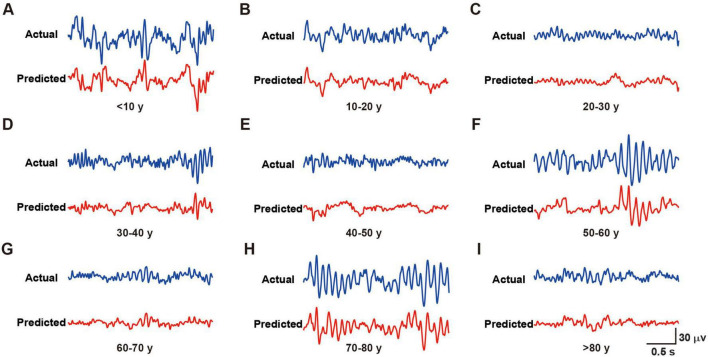
Representative actual and predicted waveforms from different age groups. **(A–I)** Blue lines represent the actual recorded waveforms, while red lines show the corresponding predicted waveforms for subjects in age groups of < 10 years **(A)**, 10–20 years **(B)**, 20–30 years **(C)**, 30–40 years **(D)**, 40–50 years **(E)**, 50–60 years **(F)**, 60–70 years **(G)**, 70–80 years **(H)**, and > 80 years **(I)**. Scale bar [shown in panel **(I)]** applies to all panels: 30 μV/0.5 s.

The RBF network demonstrated outstanding predictive performance (Figure 2), as quantified by two widely accepted statistical metrics: Normalized Root Mean Square Error (NRMSE) and Pearson correlation coefficient (r). The model achieved an NRMSE of 0.0671 ± 0.0074 on the test set, indicating minimal deviation between predicted and actual EEG signals. The Pearson correlation coefficient of 0.7209 ± 0.0678 between predicted and actual EEG signals further validated the strong positive correlation between model predictions and real signals, highlighting the practical significance of our achieved prediction accuracy.

In conclusion, the RBF neural network exhibited excellent performance in EEG data simulation and prediction, providing an effective tool for investigating the non-linear dynamics of brain electrical activity.

### 3.3 Spectral analysis evaluation of RBF network performance

**FIGURE 3:**
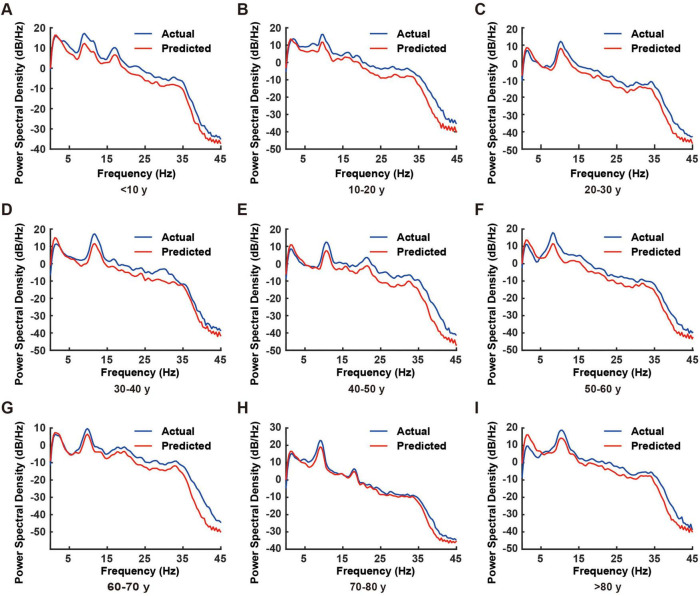
Comparison of power spectral density between actual and predicted electroencephalogram (EEG) signals across different age groups. **(A–I)** Representative power spectral density plots from individual subjects in different age groups (< 10, 10–20, 20–30, 30–40, 40–50, 50–60, 60–70, 70–80, and > 80 years). Blue lines represent actual EEG recordings, while red lines indicate model-predicted EEG signals. The power spectral density was calculated over the frequency range of 0–45 Hz.

This study employed spectral analysis to evaluate the RBF neural network’s prediction capabilities across different frequency bands, comparing actual EEG signals with predicted outputs. Figure 3 illustrates the power spectral density (PSD) comparison between these signals across four standard EEG frequency bands: δ (0.5–4 Hz), θ (4–8 Hz), α (8–13 Hz), and β (13–30 Hz).

The spectral analysis demonstrated exceptional prediction performance of the RBF network across all frequency bands (Figure 3). The PSD curves of predicted and actual EEG signals showed remarkable concordance, with nearly 100% matching in the δ and θ bands. Although minor deviations were observed in the α and β bands, the predicted signals maintained accurate representation of the primary spectral characteristics.

This high prediction accuracy was consistently observed across all nine age groups, validating the RBF network’s superior generalization capability. The network model, trained on 40,000 data points, demonstrated robust adaptation to EEG characteristics across different age groups, confirming its reliability.

Notably, while prediction accuracy showed slight degradation in higher frequency bands compared to lower frequencies, attributable to the inherent complexity and variability of high-frequency neural activity, the model successfully captured key features in the α band, establishing a foundation for future research in high-frequency EEG analysis.

### 3.4 Time-frequency analysis assessment of RBF network performance

We conducted time-frequency analysis using Short-Time Fourier Transform (STFT) to evaluate the RBF network’s capability in capturing EEG signal dynamics. The analysis employed a Hamming window function (256-point window length, 128-point overlap, 512-point FFT), generating spectrograms that enabled direct comparison between actual and predicted signals across the 0–50 Hz frequency range.

**FIGURE 4:**
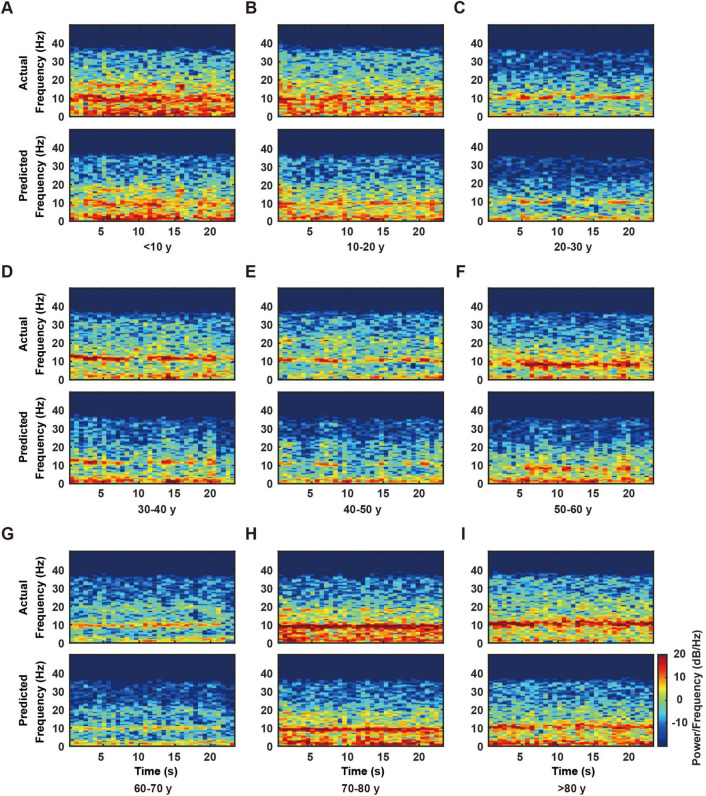
Time-frequency analysis of actual and predicted electroencephalogram (EEG) data across different age groups. **(A–I)** Representative time-frequency plots from individual subjects in nine age groups (< 10, 10–20, 20–30, 30–40, 40–50, 50–60, 60–70, 70–80, and > 80 years). For each age group, the upper panel shows the actual EEG time-frequency distribution, while the lower panel displays the corresponding model-predicted time-frequency distribution. The color scale, shown on the right side of panel I and applicable to all panels, represents power/frequency (dB/Hz). Note the consistency between actual and predicted patterns across all age groups, particularly in the lower frequency bands (0–20 Hz).

Results demonstrated the RBF network’s successful reconstruction of primary frequency components and their temporal evolution (Figure 4). Specifically, both actual and predicted signals exhibited consistent patterns across multiple frequency bands: sustained high-energy distributions in the delta band (0–4 Hz), accurate capture of periodic power modulations in the alpha band (8–13 Hz), and precise representation of intermittent activities in the beta band (13–30 Hz).

The time-frequency analysis results validated the high concordance between predicted and actual signals in both frequency distribution and temporal evolution. These findings, in conjunction with previous NRMSE and spectral analyses, substantiate the RBF network’s reliability and accuracy in reconstructing EEG signal dynamics.

### 3.5 Age-dependent analysis of RBF network fixed-point spatial coordinates

**FIGURE 5:**
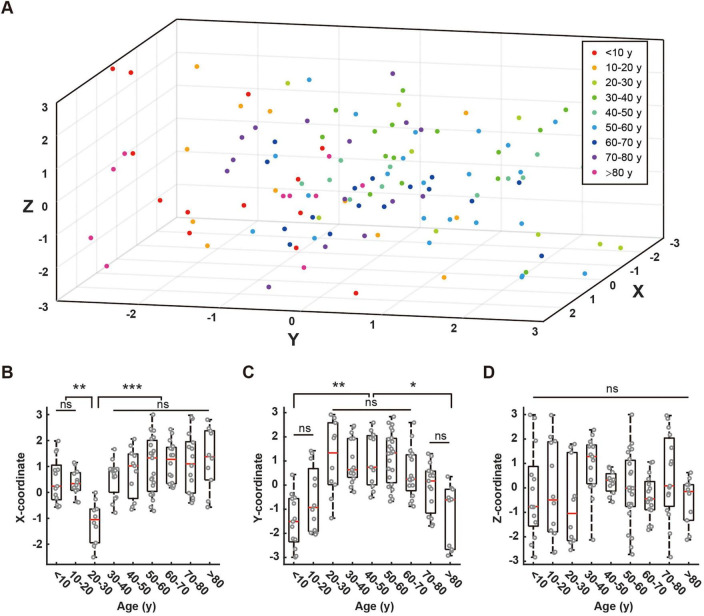
Distribution of fixed points across different age groups and their coordinate comparisons. **(A)** Three-dimensional scatter plot showing the distribution of fixed points for all age groups. Different colors represent different age groups: < 10 years (red), 10–20 years (orange), 20–30 years (light green), 30–40 years (dark green), 40–50 years (green), 50–60 years (light blue), 60–70 years (blue), 70–80 years (purple), > 80 years (pink). **(B–D)** Box plots comparing the distribution of fixed points along X-axis **(B)**, Y-axis **(C)**, and Z-axis **(D)** across age groups. Red lines in box plots indicate means. Statistical significance is denoted as follows: ns (not significant, *p* > 0.05), * (*p* < 0.05), ** (*p* < 0.01), *** (*p* < 0.001).

We investigated the relationship between age and three-dimensional spatial coordinates (X, Y, Z) of RBF network fixed points. After excluding 17 participants with unstable equilibrium points (41.2% under age 10), distinct age-related patterns emerged across spatial dimensions (Figure 5A).

The X-coordinates showed minimal values in the 20–30 age group, with notable negative values (ANOVA, 10–20 vs. 20–30, *P* = 0.0064; 20–30 vs. 30–40, *P* = 0.0005), while maintaining stability across other age groups without significant between-group differences (Figure 5B). The Y-coordinates exhibited a non-linear age-related pattern (Figure 5C). Values were initially low in the < 10 age group (Kruskal-Wallis, < 10 vs. 20–30, *P* = 0.0021), increased during ages 10–20, and stabilized between ages 20–60 (Kruskal-Wallis, 20–30 vs. 50–60, *P* = 1.0000). A declining trend emerged after age 60, with significant reductions observed in the > 80 age group compared to middle-aged adults (Kruskal-Wallis: 60–70 vs. > 80, *P* = 0.0013). The most pronounced decline was evident in octogenarians. The Z-coordinates displayed variations across age groups but lacked significant age-related trends or inter-group differences (ANOVA, *P* = 0.1352; Figure 5D).

These results reveal complex age-dependent patterns in spatial coordinates, with each dimension exhibiting distinct characteristics, thereby offering new insights into age-related EEG features.

### 3.6 Performance comparison with alternative models

To further validate the effectiveness of the RBF network, we compared its performance against three commonly used architectures in EEG analysis: Multilayer Perceptron (MLP), Convolutional Neural Networks (CNN), and Long Short-Term Memory (LSTM) networks. As summarized in Table 2, the RBF network achieved an NRMSE of 0.0671 ± 0.0074 and a Pearson correlation coefficient (r) of 0.7209 ± 0.0678, which is comparable to CNN (0.0614 ± 0.0068, r = 0.7512 ± 0.0621) and LSTM (0.0589 ± 0.0072, r = 0.7689 ± 0.0597) while requiring significantly lower computational resources. In contrast, the MLP model exhibited lower predictive accuracy (NRMSE = 0.0893 ± 0.0105, r = 0.6583 ± 0.0732), likely due to its limited capacity to capture EEG signal dependencies. Training time was significantly shorter for the RBF network (40 min) compared to CNN (120 min) and LSTM (150 min), and inference was nearly instantaneous (< 0.01 s per trial), making it a computationally efficient option. Given these results, the RBF network provides a practical balance between accuracy and computational feasibility, making it well-suited for EEG-based dynamical system reconstruction and real-time applications.

**TABLE 2:** Performance comparison of different neural network models for electroencephalogram (EEG) analysis.

Model	NRMSE (Mean ± SD)	Pearson correlation (r)	Training time (minutes)	Inference time (per trial, s)	Computational resource requirement
RBF network	0.0671 ± 0.0074	0.7209 ± 0.0678	40	< 0.01	Low
MLP	0.0893 ± 0.0105	0.6583 ± 0.0732	55	< 0.01	Medium
CNN	0.0614 ± 0.0068	0.7512 ± 0.0621	120	0.05	High
LSTM	0.0589 ± 0.0072	0.7689 ± 0.0597	150	0.08	High

CNN, convolutional neural network; LSTM, long short-term memory; MLP, multilayer perceptron; NRMSE, Normalized Root Mean Square Error; RBF, radial basis function.

## 4 Discussion

In this investigation, we developed an innovative framework for EEG signal analysis by combining RBF neural networks with Particle Swarm Optimization (PSO) to reconstruct and analyze brain system dynamics. This approach demonstrates both predictive accuracy for system trajectories and powerful insights into the underlying dynamical properties of complex neural systems.

To ensure computational efficiency while maintaining high predictive accuracy, we optimized both hardware usage and algorithmic efficiency. All experiments were conducted on an Intel Core i7-12700K CPU with 32GB RAM and an NVIDIA RTX 4070 GPU. Training the RBF network on the full dataset of 142 participants took approximately 40 min, while the inference phase was nearly instantaneous (< 0.01 s per trial), making it suitable for real-time applications. Although RBF networks can become computationally expensive with an excessive number of neurons, we employed a controlled neuron allocation strategy using principal component analysis (PCA)-guided feature reduction to optimize model complexity while preserving performance. Additionally, batch processing and parallel computation in MATLAB were implemented to reduce memory overhead during training. These optimizations allowed our approach to remain scalable without requiring excessive computational resources. Future work could explore sparse RBF architectures or hardware-specific optimizations such as GPU-accelerated training to further improve efficiency for larger EEG datasets.

Our results indicate that RBF networks effectively capture the essential characteristics of EEG data, particularly excelling in spectral reconstruction. While minor variations in frequency energy distributions were observed, attributable to both the inherent complexity of neural signals and training data limitations, these findings suggest potential improvements through expanded datasets and enhanced computational resources, albeit at increased computational cost.

Utilizing the trained RBF network, we successfully employed PSO algorithms (Gad, 2022) to identify system fixed points, establishing critical markers for understanding neural dynamics. In the context of dynamical systems theory, these fixed points serve as fundamental determinants of both local and global system behavior (Bollman et al., 2007; Parmananda, 2003). This methodology provides a robust framework for analyzing the nonlinear dynamics inherent in EEG systems, offering new perspectives in neurological research.

In our investigation of age-related characteristics, several significant findings emerged: First, Y-axis values demonstrated non-linear age-dependent changes, with notably lower values in individuals under 20 and over 70 years of age, while maintaining stability between 20 and 70 years. This pattern strongly correlates with established patterns of brain development and aging, reflecting dynamic functional changes across the lifespan. Particularly noteworthy was the upward trend observed in the 10–20 age group, which, although not significantly different from the 0–10 age group, likely indicates crucial developmental features during adolescence.

We observed a significant decrease in X-axis values around age 20–30, potentially indicating unique patterns of brain functional organization in young adults, possibly linked to synaptic pruning and neural network optimization. The Z-axis showed no significant variations across age groups, suggesting that certain fundamental neural activity patterns remain stable throughout the life cycle.

Importantly, the 0–10 age group frequently failed to achieve equilibrium points, which may be attributed to two factors: data quality issues due to poor test compliance in young children, or the inherent dynamic instability characteristic of the developing brain, consistent with rapid neurodevelopment and plasticity during this period. These findings suggest new directions for clinical EEG applications, particularly in the early detection and intervention of age-related cognitive disorders and neurodegenerative diseases. These findings demonstrate that age-related neuroplasticity changes are intimately linked to specific synaptic functions and neural network connectivity, providing novel perspectives for understanding cognitive decline in aging populations. This work presents new approaches for EEG signal analysis and early diagnosis of age-related neurological disorders, while deepening our understanding of neurodegenerative diseases and potentially informing the development of targeted therapeutic strategies to improve associated cognitive dysfunctions.

The findings of this study have important implications for clinical EEG analysis and neurological disorder assessment. The RBF network’s ability to reconstruct EEG dynamics and extract age-related fixed-point characteristics suggests potential applications in early detection and monitoring of neurodegenerative diseases such as Alzheimer’s and Parkinson’s disease, where EEG abnormalities often emerge before clinical symptoms. Additionally, the model’s computational efficiency and real-time inference capability make it suitable for ICU EEG monitoring, cognitive function assessment, and brain-computer interface (BCI) applications. Its ability to analyze neural activity dynamically without extensive computational resources enhances its feasibility for bedside monitoring and mobile EEG devices. Future studies could explore multi-modal EEG-fMRI integration and clinical dataset validation to further expand its translational potential in neurological and cognitive disorder diagnosis.

The selection of the RBF neural network in this study was guided by several key considerations that make it particularly suitable for EEG signal analysis. While deep learning architectures such as CNNs and LSTM networks have demonstrated strong performance in various EEG classification tasks, their applicability to dynamical system reconstruction and fixed-point analysis remains limited. EEG signals exhibit complex nonlinear and non-stationary properties, requiring models capable of efficiently capturing these dynamics. RBF networks serve as universal function approximators, providing a smooth and interpretable representation of EEG dynamics (Zhou and Li, 2020). Unlike deep learning models, which often function as “black boxes,” RBF networks allow for direct interpretation of the learned transformation, which is particularly valuable in neuroscience research. Additionally, deep learning models such as CNNs and LSTMs typically require large datasets for optimal performance, which poses a challenge in EEG studies where data availability is often limited. RBF networks, on the other hand, are well-suited for smaller datasets, as they require fewer training samples while still maintaining high predictive accuracy (Shorten and Murray-Smith, 1996). Moreover, training deep learning models often demands extensive computational resources and hyperparameter tuning, whereas RBF networks can efficiently converge with minimal tuning, making them a more practical choice for clinical EEG applications. The primary objective of this study was to reconstruct the underlying dynamical properties of EEG signals and analyze fixed points in brain activity across different age groups. RBF networks provide a natural framework for approximating complex continuous functions, allowing for the extraction of stable fixed points using the Particle Swarm Optimization (PSO) algorithm (Rani and Victoire, 2018). Deep learning models, while effective in classification tasks, are not inherently designed to identify and analyze such fixed points, making them less suitable for our specific research objectives. These factors collectively justify the use of RBF networks in this study, and the results obtained further confirm that RBF networks effectively capture the underlying neural dynamics of EEG signals while providing a computationally efficient and interpretable approach for analyzing age-dependent brain activity patterns.

However, several limitations must be acknowledged: First, despite the universal approximation capability of RBF neural networks (Cybenko, 1989; Hornik et al., 1989), our current RBF architecture is relatively simple and may not fully capture all features of complex EEG systems. Second, the limited sample size (*N* = 100) and uneven age distribution may affect statistical power. Additionally, batch effects in EEG data acquisition may introduce systematic biases. Future research directions should: (1) implement more sophisticated network architectures with larger training datasets; (2) address dimensional decoupling challenges; (3) conduct larger-scale cohort studies; and (4) incorporate clinical assessment metrics to enhance model accuracy and clinical relevance.

In conclusion, this study demonstrates the promising application of RBF neural networks in EEG data analysis, successfully capturing complex non-linear features of brain activity across age groups. These findings provide crucial evidence for understanding age-related changes in EEG signal dynamics, contributing to both early diagnostic strategies and therapeutic intervention approaches for neurological disorders, while establishing a foundation for future neuroscientific research.

## Data Availability

The original contributions presented in this study are included in this article, further inquiries can be directed to the corresponding authors.
